# Musculoskeletal Disorders among Italian Dentists and Dental Hygienists

**DOI:** 10.3390/ijerph18052705

**Published:** 2021-03-08

**Authors:** Maria Giovanna Gandolfi, Fausto Zamparini, Andrea Spinelli, Alessandro Risi, Carlo Prati

**Affiliations:** 1 Dental School, Department of Biomedical and Neuromotor Sciences, University of Bologna, 40125 Bologna, Italy; fausto.zamparini2@unibo.it (F.Z.); andrea.spinelli4@unibo.it (A.S.); carlo.prati@unibo.it (C.P.); 2 Program in Ergonomics, Posturology and Yoga therapy for the Degree Course in Dentistry and for the Degree Course in Dental Hygiene, School of Medicine, University of Bologna, 40125 Bologna, Italy; 3 Program in Yoga Therapy for the Specialization Course in Sports Medicine, School of Medicine, University of Bologna, 40125 Bologna, Italy; 4 Unit of Occupational Medicine, University of Bologna, 40127 Bologna, Italy; alessandro.risi@unibo.it

**Keywords:** work-related musculoskeletal disorders (WMSD), dental professionals, dentists, dental hygienists, Nordic musculoskeletal questionnaire (NMQ), working habits, physical activity, functional mobilization, dental ergonomics, posturology

## Abstract

Dental professionals often perform physically and mentally demanding therapeutical procedures. They work maintaining muscular imbalance and asymmetrical positions for a long time. The aim of the study was to describe the prevalence and the factors associated to work-related musculoskeletal disorders (WMSD) among Italian dental professionals and the most affected body regions. A cross-sectional observational study was conducted between March 2019 and February 2020. The Nordic Musculoskeletal questionnaire (NMQ) was implemented with questions related to working habits (dental occupation, working hours per week and per days, years of work) and lifestyle (practiced physical activity, including frequency and duration, mobilization activities, and knowledge of ergonomic guidelines) was used. The-chi square test was carried out to detect any statistically significant difference (*p* < 0.05). Logistic regression was carried out to detect the most significant factors associated to WMSD occurrence. A total of 284 questionnaires have been used for the analysis. A high proportion of dental professionals (84.6%) were affected by WMSD in the last 12 months. A higher prevalence was found in females (87%) when compared to males (80%). The prevalence of WMSD was correlated to the working hours/day and hour/week, with a higher risk for operators working >5 h/day and >30 h/week. In addition, a high prevalence was found in operators working for 2–5 years after graduation. Most of the surveyed dental professionals practiced physical activity (70.1%) but only a few had satisfactorily knowledge of ergonomic guidelines (12.7%). Interestingly, participants who practiced yoga or stretching as physical activities demonstrated lower WMSD (77%) when compared to other physical activities (84%). We can highlight that generic physical activities have no functional effect on WMSD for dental professionals. The most affected body areas were neck (59.9%), shoulders (43.3%), lumbar region (52.1%), dorsal region (37.7%) and wrists (30.6%). Considering the magnitude of the problem, there is an urgent need to implement the education in ergonomics among dental professionals, that may be achieved by teaching biomechanics, posturology and integrative functional therapies (such as yoga) during the university education and by promoting holistic health of dental operators.

## 1. Introduction

The dental profession is a physically and mentally demanding job due to the many therapeutical procedures, concentration and mental pressure [1].

Dental care professionals operate in a very small (and often uncomfortable) space of the mouth performing precise and time-consuming procedures. They are forced to work maintaining muscular imbalance and asymmetrical positions for a long time. Neck inclination/rotation, forward bending with loss of cervical and lumbar lordosis and raised arms working in prolonged static isometric/eccentric contraction represent the main risk factors for musculoskeletal disorders (MSD) [2].

Moreover, with the onset of the Sars-Cov2 pandemic, the use of additional protective devices increases the mental stress, the risks of imbalanced postures and further reduces the freedom of movements [3].

MSD are defined by the World Health Organization (WHO) as a disorder of the muscles, tendons, peripheral nerves or vascular system not directly resulting from an acute or instantaneous event [4].

Painful disorders and algic symptoms concerning the musculoskeletal apparatus deriving from movements in work activities are indicated as work-related musculoskeletal disorders (WMSD)**.** These musculoskeletal issues are related to continual repetition, often in a forceful manner and with a lack of recovery time [5].

WMSD among dental professionals include injuries in muscles, tendons, joints, cartilage, nerves, ligaments and the vertebral column. The unsuitable body positions while working lead to pain, spasms, joints rigidity, shivers, and disturbances in the daily life of dentists, with development of tiredness, tingling, pain and numbness in shoulders, lower back and neck. 

The etiology of occupational diseases is studied in Ergonomics (from Greek “nomos” meaning rule and “ergon” meaning work). This discipline proposes recommendations for a work organization to permit the development of the best abilities of the person. The application of precise rules and precautions contained within the ergonomics guidelines are essential to avoid the onset of WMSD in dental professionals [6,7,8]. 

Even when the ergonomic working position is maintained, dental professionals are submitted to mental and physical stress, related to repetitive movements, vibrations, static and awkward postures, excess of pressure in muscles and joints (creating mechanical failure local ischemia and energy metabolism disturbance with following muscles myalgia, spinal disk herniation and the generation of rotator cuff impingement).

The literature reports that WMSD affects dental professionals at least once in a lifetime with a prevalence ranging from 64% to 93% [9], and involves mainly the neck, lumbar region and shoulders [10,11].

The aim of the study was to describe the prevalence and the factors associated to work-related musculoskeletal disorders (WMSD) among Italian dental professionals. No previous investigations were performed in Italy, and little is known regarding the most affected body areas or of which factors result in significant association to WMSD occurrence. The null hypothesis is that WMSD occurrence significantly increases with work activities and that it is not related to the working occupation. 

Being based in an educational academic institution, involved in the formation of dental professionals, the rationale of the study is strictly correlated to spread the awareness of the WMSD incidence and the importance of its prevention, and to schedule adequate ergonomic training for undergraduate students and courses in ergonomics for graduate dental professionals and to provide necessary knowledge in dental ergonomics.

## 2. Materials and Methods

This is a cross-sectional observational study conducted between March 2019 and February 2020. 

The study took into consideration dental professionals, namely dentists (comprising general practitioners and dental specialists) and dental hygienists. The recruitment was performed during 7 conferences and dental meetings throughout Italy. 

Two operators delivered the questionnaires by hand distribution, explaining the filling methodology to each participant.

Questionnaires were given individually and filled anonymously. To avoid errors and misunderstandings during questionnaire compilation, participants were encouraged to complete in its entirety and any explanation was given. 

Dental professionals willing to participate had to fulfill the following inclusion criteria: a stable occupation in the dental profession, a degree in dentistry or dental hygiene and age comprised between 20 and 70 years. Exclusion criteria were the following: retired professionals, undergraduate students, any major accidents, traumas or medical conditions that lead to musculoskeletal permanent disabilities.

Uncomplete questionnaires were excluded.

Questionnaires were also provided to private dental offices and to postgraduate students enrolled in the master program of the Dental School at the University of Bologna, Modena and Ferrara.

An identification code referring to the place and the date of compilation was assigned to each questionnaire. 

The approval and ethical review of the procedures and methodologies for collecting data of survey-based research were provided by the Decane of University of Bologna School of Dentistry; the survey was agreed by the Person in charge of each Organization where questionnaires were distributed. The standard Nordic Musculoskeletal Questionnaire (NMQ) [12] was translated into the Italian language [13] by the authors and has been edited and implemented with some modifications. The standard NMQ included just the musculoskeletal issues, identifying areas of the body and the functional impact of the disorders at home and work. The implementations were made to adapt the NMQ to the dental profession but the structure of the questionnaire was not distorted and its main characteristics (standardization and reproducibility) were not lost. 

Questions concerning the main working activity, lifestyle and knowledge of ergonomics guidelines were added, namely:

- Main outpatient work of the operators: dental specialists (i.e., endodontics, oral surgery, prosthetics, conservative, orthodontics, periodontics); general practitioners and dental hygienists;
- Practiced physical activities, including frequency (times per week) and duration (hours per day);
- Knowledge of the ergonomics guidelines and their application.

The questionnaire consisted of 3 parts with a total of 131 multiple choice questions.

To correctly complete the NMQ, a duration of approximately 10 min was calculated.

### 2.1. Socio-Demographic Description of the Operators

This first part of the questionnaire was the only section modified trough the addition of specific questions related to working habits, lifestyle and knowledge of ergonomics guidelines.

Therefore, this part was divided in the following sections:

- General standard information, i.e., gender, age and height (from the NMQ). Weight information was replaced by the authors with a question on self-reported body fatness [14,15] and the following parameters were implemented by authors in the NMQ, namely:
- Dental occupation and working information added by the authors in the NMQ (working hours per day, working hours per week and years of work);
- Physical activity comprising walking, running, swimming, body building, stretching, Pilates and yoga and other activities, including their frequency and duration;
- Presence of a medically diagnosed MSD;
- Knowledge and application of ergonomic guidelines.

### 2.2. MSD Prevalence and Affected Body Areas

Following the standard NMQ, the WMSD of the surveyed dental professionals were collected in this part. The presence of musculoskeletal symptoms has been considered as WMSD.

Questions about the presence of painful symptomatology during the last 12 months and 7 days in one of 9 body regions examined (neck, shoulder, elbows, wrist/hands, dorsal region, lumbar region, knees, ankle/feet) were present. 

### 2.3. Disability Due to WMSD According to Dental Work

In part 3, only WMSD that temporarily hindered or modified dental work in the last 12 months were recorded. This part was divided into nine sections, each of these containing one body region.

### 2.4. Statistical Analysis

An Excel document was used to collect the data. The questionnaires were inserted along the rows of the excel page. Each row corresponded to a surveyed dental operator, while each question corresponded to a column. The possible answers for each question were identified as a number (for example: yes = 1, no = 2).

SPSS software (IBM Corp, Amonk, NY, USA) was used for statistical analysis. Only questionnaires which were complete in their entirety were used. Partially incomplete questionnaires were discarded.

Chi-square tests were used to examine the differences of WMSD prevalence between each variable. The *p*-value was set to 0.05.

Linear logistic regression was performed to examine the factors that may be related to the occurrence of WMSD. The following parameters were considered, namely gender, age, self-reported body fatness, height, dental occupation, working hours per day, working hours per week, years of work, physical activity, physical activity duration, physical activity frequency, mobilization activities and ergonomic knowledge. The *p*-value was set to 0.05.

## 3. Results

### 3.1. Socio-Demographic Description of the Operators

A total of 323 questionnaires were distributed. Out of these, 12 were incorrectly filled and 27 were given back unfilled. A total of 284 questionnaire were useful for the analysis.

**Figure 1:**
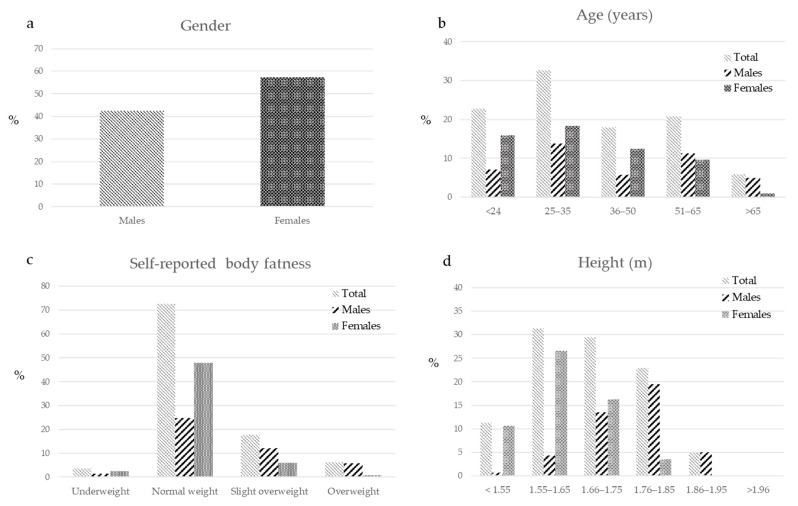
Demographic distribution of the surveyed operators, expressed in percentages. (**a**) A higher percentage of females was found (57%). (**b**) Age of the operators was highly variable and comprises a high percentage of young dental professionals. In particular, over 50% of the participants were younger than 35 years (**c**) Over 70% of the surveyed professionals had normal weight and approximately 20% stated slight overweight. (**d**) The height was uniformly distributed with small groups of very tall and very short operators.

Concerning the gender, a slightly higher presence of females was present (57%), compared to males (43%) (Figure 1a).

The age range showed a prevalence of young dental professionals, with age < 35 years (Figure 1b).

Over 70% of the surveyed dental professionals reported a normal body fatness (Figure 1c).

Most of the interviewed dental professionals were between 1.55 and 1.85 m height, namely distributed in 3 groups uniformly represented (30% each) (Figure 1).

Female participants presented with heights < 1.75 m, with a markedly higher percentage of <1.55 m compared to males. By contrast, males showed height comprised between 1.66 and 1.95 m.

**Figure 2:**
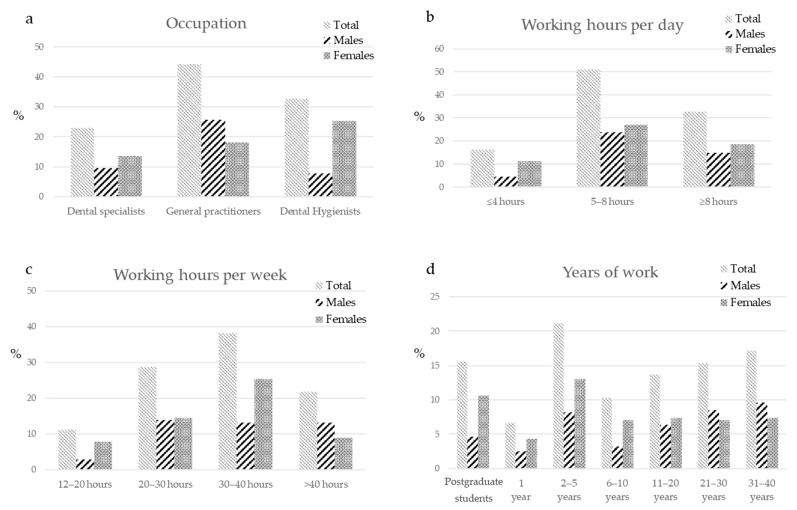
Occupational distribution of surveyed operators, expressed in percentages. (**a**) The population was constituted by a higher percentage of general practitioners (approximately 50%), followed by dental hygienists (approximately 30%) and dental specialists (approximately 20%). (**b**) Most of the operators worked more than 5 h per day and (**c**) more than 30 h/week. (**d**) The years of work was uniformly distributed.

As regarding the main working occupation, most of the surveyed operators were general practitioners and dental hygienists (Figure 2a).

Dental specialists, i.e., operators working in only one discipline were the least represented. Dental specialists group included endodontists (29.2%), restorative dentists (23.1%), prosthodontists (12.3%), periodontists (10.2%), oral surgeons (8.6%), orthodontists (8.7%) and implantologists (7.5%).

Most of the surveyed personnel worked 5–8 h per day (approximately 50%) or more than 8 h per day (over 30%) (Figure 2b).

Very few surveyed operators worked less than 20 h per week (approximately 10%), most of the operators worked more than 30 h per week (Figure 2c).

The number of years of work is uniformly distributed among dental professionals (Figure 2d).

**Figure 3:**
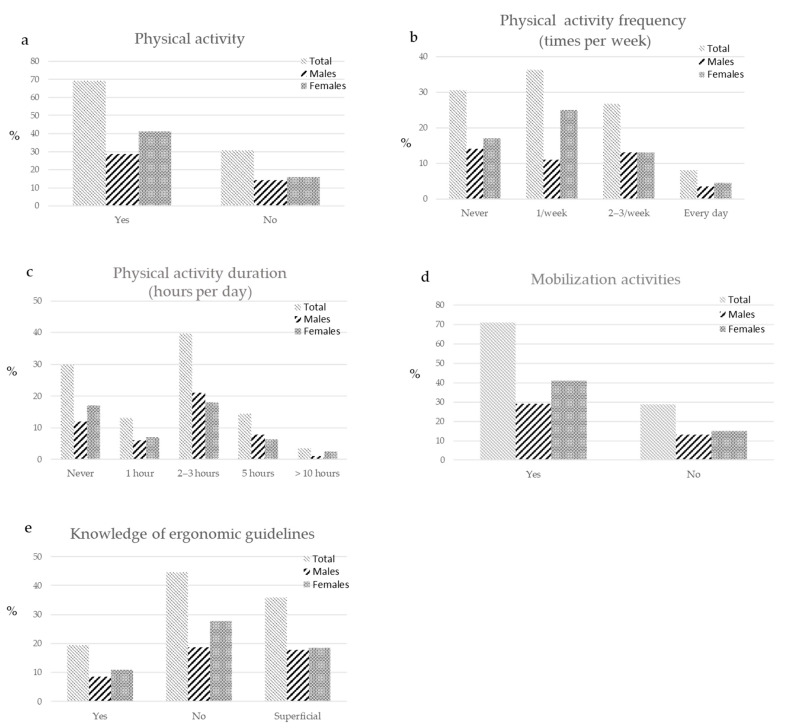
Lifestyle and knowledge of ergonomics. Practice of physical activity, including frequency and duration, mobilization activities and ergonomic knowledge of surveyed operators, expressed in percentages. (**a**) The image shows that a very low percentage of operators (approximately 30%) did not practice physical activity. The physical activity was performed mostly (**b**) 1 time a week and (**c**) 2–3 h per week. (**d**) A high number of dental professionals performed some mobilization activities (approximately 70%). (**e**) Few operators (only 20%) declared a complete knowledge of the ergonomic guidelines, a total 35% had a superficial knowledge and more than 40% of surveyed operators did not know the guidelines.

Physical activity was performed by a high percentage of dental professionals (Figure 3a), while no physical activity was performed by approximately 30% of surveyed operators (Figure 3b). 

The most reported physical activity frequency and duration were 1 time per week and 2–3 h per week (Figure 3c). Operators declared to perform some mobilization activity (approximately 70%) (Figure 3d). 

Many of the surveyed operators superficially know (approximately 35%) or do not know (over 40%) the ergonomic guidelines, while few operators had a satisfactorily knowledge (Figure 3e). 

### 3.2. WMSD Prevalence vs. Examined Parameters

A total of 241 participants (84.9%) reported a WMSD in the past 12 months. Of these, 202 surveyed operators (83.1%) declared a medically diagnosed MSD. WMSD that hampered daily routine in the last 12 months was observed in 89 participants (30.1%). A total of 122 operators (42.9%) experienced WMSD in the last 7 days (Table 1). 

Table 2 reports the prevalence of WMSD in relation to demographic parameters. 

The percentage of WMSD increased with age, dental professionals under 35 years showed lower percentages when compared to older dental professionals, with a peak between 36–50 years (94.2%).

Self-reported body fatness did not influence the prevalence of WMSD.

Tall operators showed increased prevalence of WMSD and short operators reduced WMSD.

Females showed a significantly higher percentage of WMSD when compared to males in all the evaluated parameters (*p* < 0.001).

The prevalence of WMSD according to occupation is reported in Table 3 Dental specialists showed markedly higher percentages of WMSD-affected operators (90.8%) when compared to general practitioners (84.9%) and dental hygienists (80.7%).

Higher working hours per day and working hours per week demonstrated a greater percentage of WMSD. In particular operators working more than 8 h a day and more than 40 h per week had one or more WMSD in 90% of the cases.

The years of work showed to increase the percentage of WMSD, with the highest percentages after 10–20 years of work (94.9%). Similarly, 90% of the operators working since 30–40 years showed WMSD (89.8%).

Females showed significantly higher percentages in relation to occupation, working hours per day and years of work (*p* < 0.05).

Table 4 reports the prevalence of WMSD according to physical activities, their frequency and knowledge of ergonomics guidelines. Operators performing physical activity every days or at least two-three times per week show slightly lower percentages of WMSD. However, no statistically significant differences in WMSD prevalence in relation to the variables were observed.

Females showed slightly higher percentage of WMSD when compared to males, these differences were not statistically significant.

Linear logistic regression is reported in Table 5. The analysis revealed that only gender and working hours per day parameters significantly affect the occurrence of WMSD, the *p*-values were 0.031 and 0.041, respectively.

### 3.3. WMSD Distribution According to Body Region

**Figure 4:**
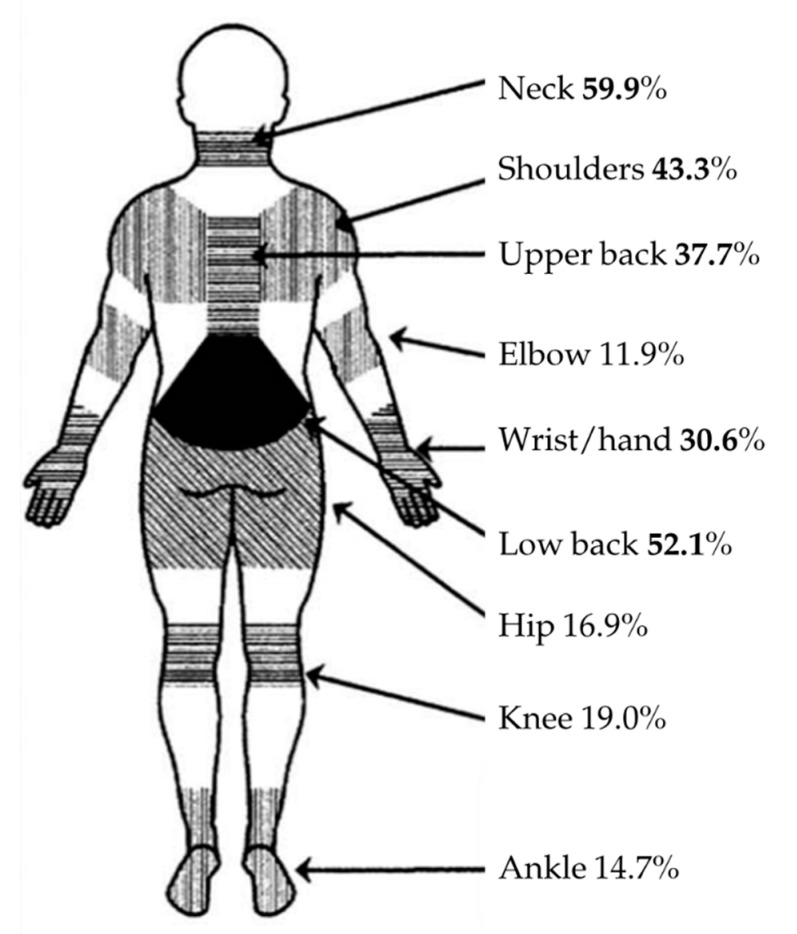
Frequency of WMSD and distribution according to body regions.

Figure 4 reports the most frequent areas where WMSD occurred. A total of 201 out of 284 operators indicated one or more WMSD. The most affected areas resulted: neck (approximately 60%), lumbar region (52.1%), shoulder (43.3%), dorsal region (37.7%) and wrist (30.6%). Elbow, hip, knee and ankle were less frequently involved.

### 3.4. WMSD vs. Working Occupation

**Figure 5:**
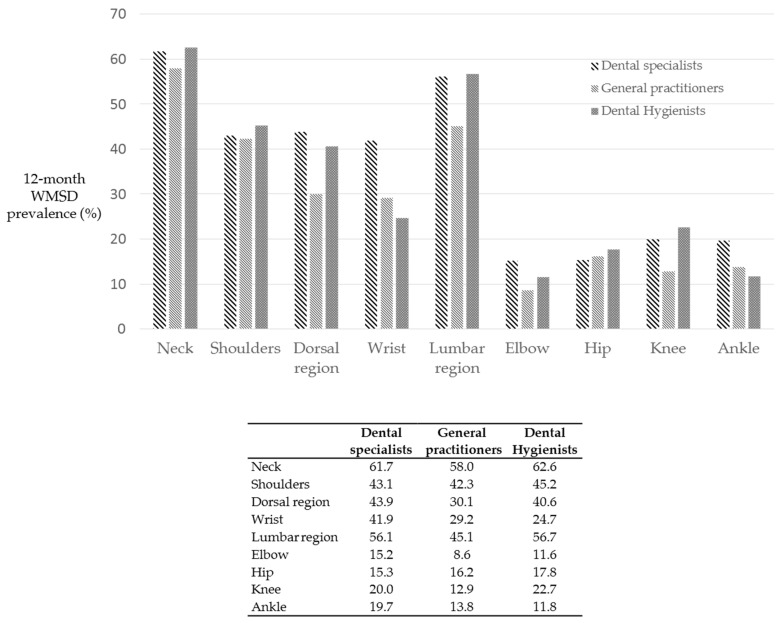
Distribution of WMSD, expressed as percentages, in relation to odontological activity. Dental specialists group included endodontists (29.2%), restorative dentists (23.1%), prosthodontists (12.3%), periodontists (10.2%), oral surgeons (8.6%), orthodontists (8.7%) and implantologists (7.5%). No significant differences were observed when considering dental occupation (*p* > 0.05).

Figure 5 reports the distribution of WMSD according to dental occupation.

All workers displayed high percentages of WMSD in the neck region (approximately 60%).

Dental specialists and dental hygienists showed similar percentages of WMSD in the neck, shoulder, dorsal region, lumbar region and alike percentages in the elbow, knee and hip. By contrast, general practitioner showed lower percentages of WMSD in dorsal region, lumbar region, elbow and knee.

**Figure 6:**
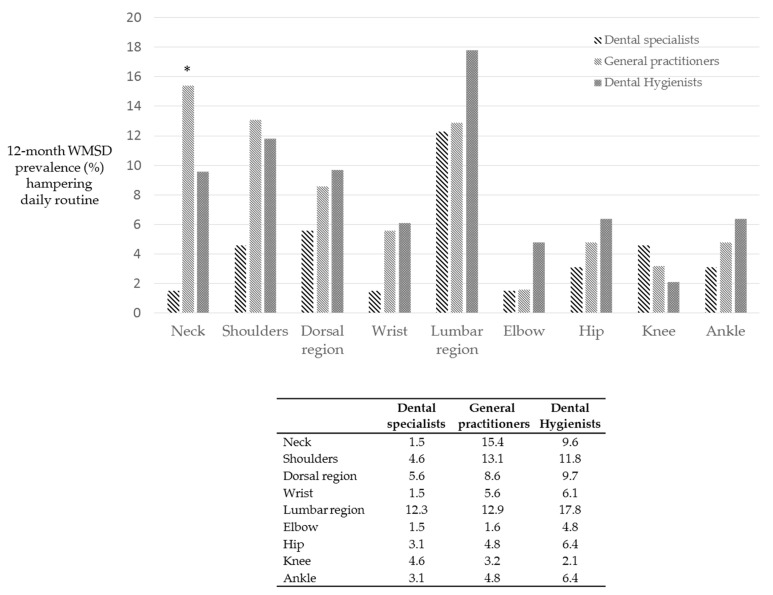
WMSD that temporarily hindered or modified dental work and daily routine in the last 12 months. Asterisk indicates statistically significant differences (*p* < 0.05).

Figure 6 reports the distribution of WMSD that temporarily impeded the normal activity and daily routine in the last 12 months. A total of 89/284 operators (approximately 30%) experienced this disability in the last 12 months. General practitioners revealed statistically higher WMSD disability percentages in the neck region (*p* = 0.035) and elbow when compared to dental hygienists and dental specialists. By contrast, dental hygienists showed markedly higher percentages of WMSD in lumbar region and elbow. Interestingly, dental specialists showed lower percentages of WMSD disabilities in almost all investigated areas, only the lumbar region showed percentages similar to general practitioners.

**Figure 7:**
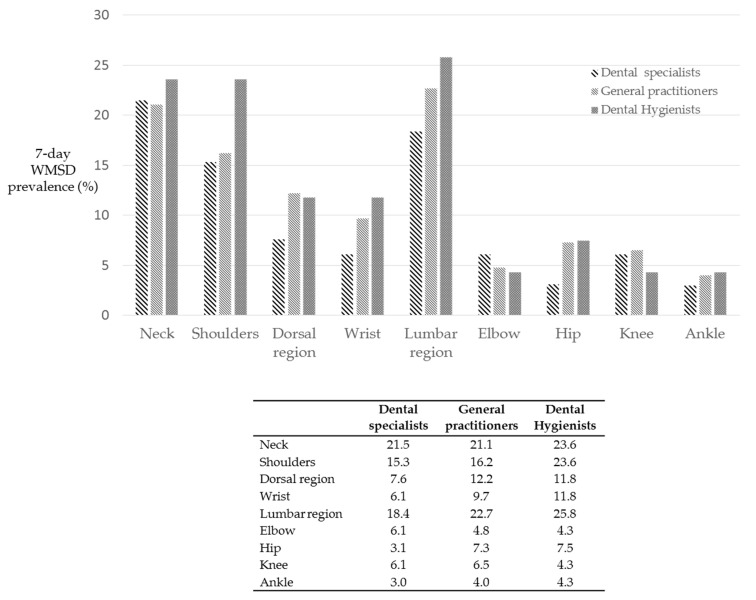
Distribution of WMSD experienced in the last 7 days by 284 surveyed dental professionals. Neck, shoulder and lumbar region were the most affected areas, involved in approximately 20% of the operators. No significant differences were observed when considering dental occupation (*p* > 0.05).

Figure 7 reports the prevalence and area distribution of WMSD which occurred in the last 7 days among 284 dental professionals. A total of 122/284 (42.9%) operators experienced WMSD in the last 7 days. Dental hygienists showed the highest presence of WMSD, in particular in neck, shoulder and lumbar region. Dental specialists showed the lowest percentages of WMSD in almost all the region areas.

### 3.5. WMSD per Gender

**Figure 8:**
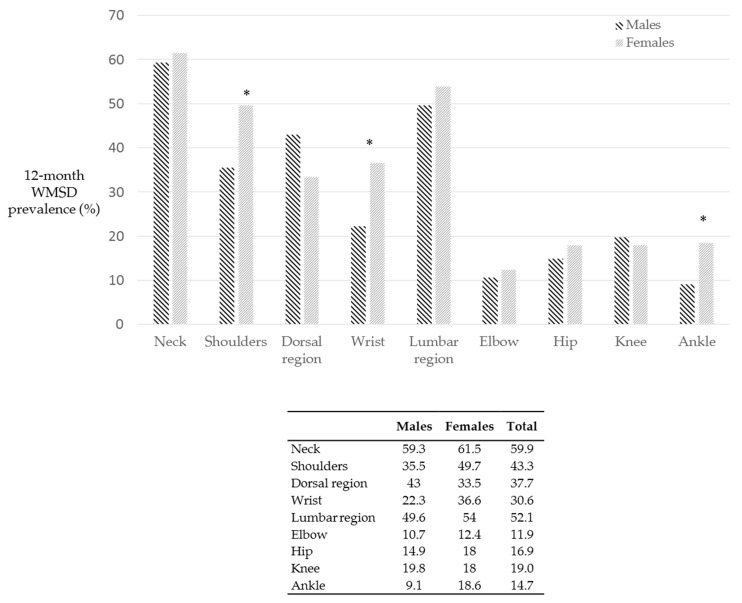
WMSD experienced in the last 12 months according to gender variables. Females showed a higher presence of WMSD in most of the body regions. Asterisks indicate statistically significant differences (*p* < 0.05).

Figure 8 reports the body area distributions of WMSD according to gender variables. Females revealed higher percentages of WMSD in the last 12 months in 7 body areas out of 9.

WMSD at the shoulders, wrists and ankles are significantly higher in females when compared to males, *p*-values were 0.43, 0.41 and 0.21. respectively. Males showed higher presence of WMSD in dorsal region, the percentages were. WMSD at the knees were slightly higher in men.

**Figure 9:**
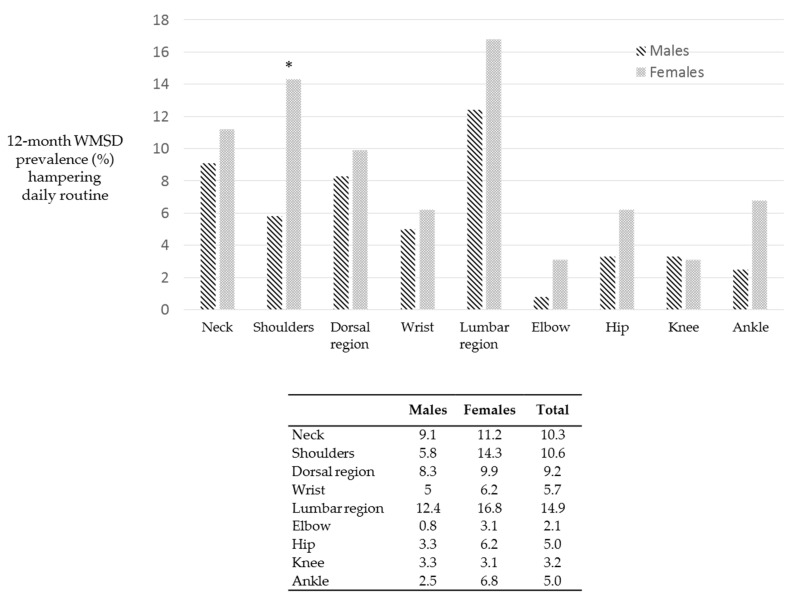
WMSD that impeded to perform normal activities in the last 12 months. Females reported higher percentages of WMSD in 8 / 9 body areas. Asterisk indicates statistically significant differences (*p* < 0.05).

Figure 9 reports WMSD that impeded to perform routinely activities in the last 12 months. Females showed higher percentages than males in 8 out of 9 body areas. Lumbar regions, shoulders and neck were the most affected areas. In particular, percentages of WMSD in the shoulder region were significantly higher in females than males (*p* = 0.028).

**Figure 10:**
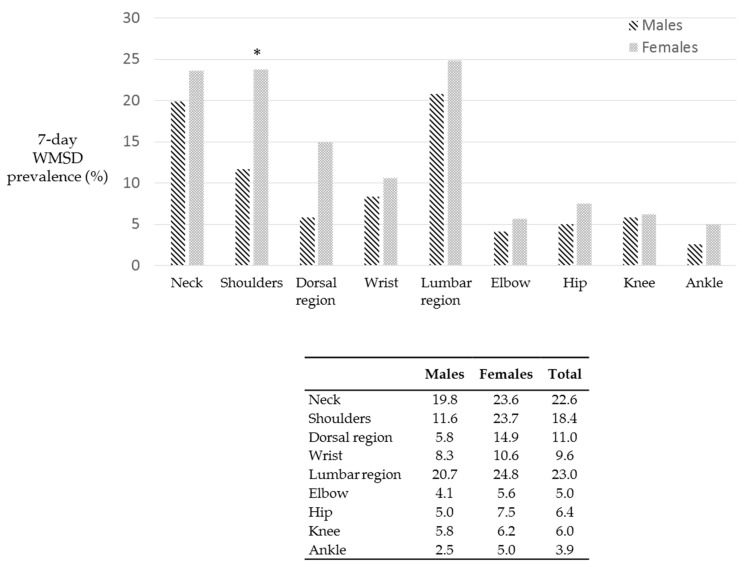
WMSD experienced in the last 7 days. Females reported higher WMSD percentages in all body areas when compared to males. Asterisk indicates statistically significant differences (*p* < 0.05).

Figure 10 reports the WMSD percentage in the last 7 days according to body region. Females showed higher WMSD percentages in all cases. The most affected areas were the Lumbar region (23.0%), neck (22.6%), shoulders (18.4%) and dorsal region.

Females showed statistically higher percentages (*p* = 0.024) in shoulder region compared to males, the values were 23.7% and 11.6% respectively. Similarly, dorsal region demonstrated statistically higher percentages (*p* = 0.038) when compared to males, the values were 14.9% and 5.8%.

## 4. Discussion

This study examined the prevalence of WMSD among Italian dental care workers, comprising dental specialists, general practitioners and dental hygienists. To the best of our knowledge, this is the first study on the Italian dental professionals.

Several articles published in last decades on the work-related musculoskeletal disorders among dental operators, showed that is a very important issue that still remains unsolved, with few solutions proposed to prevent it [9,16,17,18,19,20,21,22,23,24,25,26,27]. WMSD are very common in the population and in particular among dentists with a range from 64% to 93% [9]. 

A recent Cochrane study highlighted the lack of physical, cognitive and organizational ergonomic interventions for preventing WMSD in dental care practitioners [28].

The Nordic musculoskeletal questionnaire has been developed to assess the severity and impact of musculoskeletal symptoms in different occupational groups [9], such as health care professionals [18,29] or among industrial workers [30].

In the present study, several parameters including main dental occupation, number of worked hour per day and week, years of experience, and physical activities, performance of mobilization activity and knowledge ergonomic guidelines have been added in the standard questionnaire.

Other studies investigated the prevalence of WMSD in dentistry using a modified Nordic Musculoskeletal Questionnaire [16,20,21,22] with the addition of dental activity questions [16], some questions on physical activity [20], questions on musculoskeletal symptoms [21] or questions on pain disability and application of ergonomic activities [22].

As main result, this investigation revealed that the prevalence of WMSD in the past 12 months among the 284 participants was 84.9%.

Other previous studies showed a prevalence of 81.4% among 204 Brazilian dentists [16], of 87.2% among 285 Australian dentists, [17] of 86.5% among 2449 dentists in Lithuania [18] and of 85.6% among 288 dentists in China [24].

Other studies showed a higher prevalence, namely 94% among 120 dentists in Turkey [31], 92% among 450 dentists and dental students in Germany [16], 96% among 581 dentists in Czech Republic [23] and 95% among 80 dentists in Cameroon [22].

Only few studies showed lower prevalence of WMSD, namely 73.3% among 236 dentists in India [20], 62% among 430 dentists in Greece [27], 59.2% among 68 dentists in Saudi Arabia [19] and 42% among 390 dental students in United Kingdom [26].

WMSD hindered working activity in the last 12 months in approximately 30% of the investigated operators: 89 out of 284 dental care workers in our study referred one or more WMSD that affected their daily routine, temporarily impeding the outpatient activity. This result confirms the data from a recent review reporting that 1/3 of the dentists had to change their work activity and that 2/3 have suffered from WMSD at least once [6].

A large portion of surveyed dental professionals (approximately 40%) declared the presence of WMSD in the last 7 days. These data are lower than that reported in literature. A recent paper showed that approximately 60% of surveyed dentists experienced one or more WMSD in the last 7 days [16].

The present study confirms that the high prevalence of WMSD is due to the nature of dental work conditions and can negatively affect both the general state of health and the work activity leading to a drop in turnover and in the most serious cases to absenteeism and premature abandonment of the work.

Different risk factor stressors can be identified in dental profession. Dental activities often include forceful exertions, repetitive motions, vibration and long duration of the procedures. The workstation often includes a number of equipment and instruments that forced to maintain an incorrect posture. A prolonged and incorrect posture lead to the loss of cervical and lumbar lordosis, forward head, weak postural muscles, poor flexibility, pelvic tilt and tension in spine extensor muscles. A further support to reduce the onset of WMSD should be the redesign of dental units.

Concerning the location of WMSD, we found that the most affected regions were the neck (59.9%), followed by the lumbar region (52.1%), shoulder (43.3%) and the dorsal region (37.7%).

In the present study, the high prevalence of WMSD occurring in cervical region is in agreement with other studies [20,21].

The cervical spine is the most exposed area to develop WMSD, and mainly due to posture of dental operators working with the head tilted forward for more than 15–20°, resulting in an overload of muscles of the neck and joints of the cervical spine. In this position, posterior neck muscles (the neck extensors) work harder to hold the neck against gravity, provoking muscles tightening and leading to neck pain. This condition also affects the cervical lordosis curve. The muscles of the cervical and upper thoracic spine have to contract constantly to support the weight of the head. Loss of the cervical lordosis can cause pain, functional disabilities and disc protrusion. No differences by gender were observed when considering this body region. These data are different from that of previous studies, where females had higher percentages of WMSD in the neck than males [16,23].

WMSD in the lumbar region was present in 52.2% of the 284 surveyed dental professionals. This region was equally affected in all the 3 dental workers groups (dental specialists, general practitioners and dental hygienists). Females and males dental professionals showed comparable percentages of WMSD. A similar trend is reported in previous studies [21,22].

WMSD in the lumbar region is mainly related to the loss of the lumbar lordosis due to the incorrect sitting posture, lack of hip tilting while sitting and to the forward bent while working as well as to the relative weakness of the stabilizer muscles of the lumbar spine due to prolonged and incorrect sitting procedures.

WMSD affecting the shoulder region was present in 43.3% of surveyed dental professionals. This data is similar to the results found in previous studies [21,22]. WMSD in the shoulder region may be correlated to the forceful exertions, repetitive motions, vibration and long duration of the procedures, prolonged shoulder abduction (>45°) with elbow flexed and pronated with isometric and eccentric contraction, fatigue and strain deltoid, supraspinatus trapezius and serratus anterior. In the present study, Females showed statistically higher percentages of WMSD in the shoulder than males. These data are in accordance with a previous study [16].

The dorsal region was affected in 37.7% of surveyed dental professionals. Other studies found a similar prevalence in this region with a percentage of approximately 30% of dental professionals [24]. The WMSD in this region is related to the loss of cervical and lumbar lordosis, forward head and to prolonged seating position during work activities. In the present study, WMSD affected the wrist region in 30.6% of surveyed dental professionals. These percentages are similar to those of previous studies [16,24]. In addition, females revealed statistically higher WMSD percentages when compared to males [16], not so different from our study data.

Interestingly, in this paper, the WMSD prevalence is high in dental specialists (41.9%) and general practitioners (29.2%) and lower for dental hygienists (24.2%). Dentists specialists, general practitioners use highspeed and micromotor handpieces very frequently for removal of caries, root canal treatments, restorations and various other procedures [3]. Moreover, recurrent movements of the hand and fingers during use of hand files and broaches for root canal procedures lead to a repetitive stress. Along with prolonged gripping of the instruments the dentists have to apply considerable force while performing the above-mentioned procedures. This leads to a constant isometric contraction in the muscles leading to cumulative stress injuries. Dental hygienists use less often micromotor handpieces and more frequently ultrasonic devices for routinely scaling procedures, which may possibly explain the lower percentages of WMSD at the wrist found in the present investigation.

WMSD could develop due to multiple risks factors. Concerning the years of work, from our data it emerged that the WMSD peak is reached among those who started their work 10–20 years ago (94.9%) compared to those who have worked for 1 year (73.6%) or for more than 30 years (89.8%). This likely occurs because it is the most intense working phase of the career when the dentist is generally able to do all dental treatments. Indeed, at the beginning of the career the working activity can sometimes alternate with a phase of learning or study while in the final stage of the career the professional focuses on more complex works, with earn in terms of hours spent. For this reason, age groups have been set, operators aged <24 have been set as young post graduate professionals.

Operators aged 25–35 years (likely working for 2–5 years) as more proficient but still undergoing some education programs (e.g., master attendance, professional courses). Experienced operators aged 36–50 years (likely working for 6–20 years) and 51–65 years (likely working for 21–30 years) as likely fully dedicated to dental profession. Operators aged more than 65 years (likely working for 31–40 years) at the end of their career, likely decreasing the cumulative working hours.

Interestingly, the number of working hours per day and per week appeared significantly associated to presence of WMSD in the investigated operators (*p* = 0.027 and *p* = 0.048). Linear logistic regression confirmed the significance only of working hour per day.

The highest percentage of WMSD is for dental professionals working more than 40 h per week (88.7%) compared to those who work between 12 and 20 h per week (75.0%). Operators working many hours a day in particular without interruptions (such as short pauses or stretching breaks) favor the onset of WMSD [32,33,34]. They also have less time to dedicate to their body.

Gender results a significant variable in the present study, as confirmed by the linear logistic regression reported in Table 5.

The onset of WMSD among women is a higher (87.1%) than in men (80.2%). These data are in line with a previous study [35].

We can correlate this data with the different physical constitution and mainly with lower muscular mass and strength of the women. In addition, hormonal changes, higher incidence of osteoporosis and additional physical stress (for take care of families and children) further favors the onset of WMSD.

Although the literature highlighted the WMSD risk for dental professionals, there are still few information on the strategies to prevent WMSD.

For this reason, in this study we investigated physical activities, their duration and frequency, mobilization activities and knowledge of the ergonomic guidelines. The results clearly show a very low percentage of active operators who regularly practice physical activities (less than 10%). It is reported that physical activities have a particularly positive effectiveness in stress management and WMSD prevention [36]. In our study, 27 participants practiced yoga or stretching as physical activities. Interestingly, the WMSD prevalence in this group was 77.2%, lower than the prevalence in the whole group (84.3%). Of these, 52% declared a pre-existent MSD. We may speculate that these two disciplines may be useful to reduce WMSD occurrence. Previous studies demonstrated that yoga reduces areas of tissue restriction and induces a deep relaxation in muscle tension [37,38,39,40,41].

A high percentage (84%) of dental professionals had a partial (38%) or no (46%) knowledge of ergonomic guidelines. However, this condition is not related with WMSD onset. We may speculate that the occurrence of WMSD brought the interest to study and apply the ergonomics guidelines. It should be noted that a high percentage (approximately 75%) of WMSD is present in post-graduate students.

Students are nowadays very susceptible to WMSD. It is reported that new technologies and lack of mobility are critical factors for WMSD occurrence in young people [42]. A key role must be played by universities that must teach the correct posture and the exercises to be done to prevent WMSD in particular for the future dental professionals.

Nowadays, only few Italian university have a study plan for the degree course in dentistry that provides the teaching of ergonomics guidelines to prevent musculoskeletal disorders in the dental profession. A correct education from the beginning of the working activity could provide great benefits to dental operators who would gain in terms of health and well-being.

The majority of degree programs in dentistry and dental hygiene provide only basic ergonomic education concerning patient/operator positioning and instrumentation, but formation and training on body mechanics and preventive exercises to develop healthy working habits are unavailable with the exceptions of few university programs (we checked among the teaching programs of the top-scored world universities and Italian universities).

There is an urgent need to improve teaching dental ergonomics, through new pedagogical strategies, approaches and active learning methodologies. Students need to know and understand from the first years of university courses the knowledge of ergonomic guidelines. Through the use of new active didactic activities, students would be able to observe, learn and be aware of the correct posture, avoiding incorrect postural errors and practicing healthy attitudes that would prevent WMSD onset in dental occupation.

Limitations of the study may be that mobilization activities were not fully disclosed in the questionnaire, leading to a potential misunderstanding when referring to physical activities.

Moreover, due to the typology of the study, it is not possible to assess the benefits provided by performing mobilization activities and knowledge of ergonomic guidelines. Additionally, it is not possible to assess whether WMSD were fully attributable to the work conditions or may be attributable to pre-existent MSD. Further analyses should be performed to analyze the potentialities of mobilization activities among dental professionals and correlate the risks factors in dental practice.

## 5. Conclusions

Italian dental professionals experienced a high prevalence of WMSD.

Considering the magnitude of the problem, there is an urgent need of educational prevention at the universities providing ergonomic knowledge to dental professionals.

This consciousness in dental ergonomics may be achieved by teaching posturology, body biomechanics and integrative therapies (such as yoga) at the university and by promoting holistic health of dental operators.

Therefore, future proposals should be focused on physical and cognitive ergonomic interventions. Main efforts should involve the university degree programs towards musculoskeletal biomechanics teaching, education for preventive therapeutic mobilization and active training for detensioning and compensative exercises.

The awareness of practicing healthy attitudes and the fostering of attention to the signals and needs of the body are suggested as new pedagogical approaches to increase body and mind well-being and to improve the achievement of a pleasing long profession.

## Figures and Tables

**Table 1:** Work-related musculoskeletal disorders (WMSD) prevalence in surveyed operators (expressed as *n* and %). A high number of dental professionals (241/284) suffered from WMSD in the last 12 months. Of these, 89 operators experienced WMSD that interfere to perform daily routine in the last 12 months.

	Absence		Presence	
	*n*	%	*n*	%
WMSD in the last 12 months	43	15.1	241	84.9
WMSD hampering the daily routine in the last 12 months	195	69.9	89	30.1
WMSD in the last 7 days	162	57.1	122	42.9

**Table 2:** Prevalence of WMSD in the past 12 months according to anthropometric parameters (statistical significance for *p* < 0.05). Females showed a significantly higher percentage of WMSD when compared to males (*p* < 0.05).

			Absence of WMSD			Presence of WMSD			χ^2^ Test	
			Total	Males	Females	Total	Males	Females	Males/Females	Total/Total
		*n*	*n* (%)	*n* (%)	*n* (%)	*n* (%)	*n* (%)	*n* (%)		
Age (years)	<24	65	15 (23.1)	6 (40.0)	9 (60.0)	50 (76.9)	14 (28.0)	36 (71.0)	*p* < 0.001	*p* = 0.089
	25–35	93	16 (17.2)	10 (62.5)	6 (37.5)	77 (82.8)	29 (37.6)	48 (62.4)		
	36–50	51	3 (5.8)	1 (33.3)	2 (66.7)	48 (94.2)	15(31.2)	33 (68.8)		
	51–65	59	7 (11.9)	5 (71.4)	2 (28.6)	52 (88.1)	27 (51.9)	25 (48.1)		
	>65	16	2 (12.5)	2 (100)	0	14 (87.5)	12 (85.7)	2 (14.3)		
Self-reported body fatness	Underweight	8	2 (25)	0 (0)	2 (100)	6 (75)	1 (16.7)	5 (83.3)	*p* < 0.001	*p* = 0.898
	Normal weight	207	31 (14.9)	15 (48.3)	16 (51.7)	176 (85.1)	55 (31.2)	121 (68.8)		
	Slight overweight	51	7 (13.7)	6 (85.1)	1 (14.9)	44 (86.3)	28 (63.3)	16 (36.7)		
	Overweight	18	3 (16.6)	3 (100)	0	15 (83.4)	13 (86.7)	2(13.3)		
Height (m)	<1.55	32	7 (21.8)	1 (14.9)	6 (85.1)	25 (78.2)	1 (4)	24 (96)	*p* < 0.001	*p* = 0.613
	1.55–1.65	89	10 (11.2)	3 (30)	7 (70)	79 (88.8)	9 (11.4)	70 (88.6)		
	1.66–1.75	84	15 (17.8)	9 (60)	6 (40)	69 (82.1)	29 (42.1)	40 (57.9)		
	1.76–1.85	65	10 (15.4)	10 (100)	0	55 (84.6)	45 (84.2)	10 (15.8)		
	1.86–1.95	14	1 (7.1)	1 (100)	0	13 (92.9)	13 (100)	0		
	>1.96	0	0	0	0	0	0	0		
Total		284	43 (15.1)	24 (55.9)	19 (44.1)	241 (84.9)	97 (40.2)	144 (59.8)	*p* = 0.048	

**Table 3:** WMSD in relation to occupation, working hours per day, working hour per week and years of work (statistical significance for *p* < 0.05). The increase of working hour/day and working hour per week was significantly associated to a higher occurrence of WMSD (*p* < 0.05).

			Absence of WMSD			Presence of WMSD			χ^2^ Test	
			Total	Males	Females	Total	Males	Females	Males/Females	Total/Total
		*n*	*n* (%)	*n* (%)	*n* (%)	*n* (%)	*n* (%)	*n* (%)		
Occupation	Dental specialists	65	6 (9.2)	4 (66.7)	2 (33.3)	59 (90.8)	23 (38.9)	36 (61.1)	*p* = 0.010	*p* = 0.278
	General practitioners	126	19 (15.1)	8 (42.1)	11 (57.9)	107 (84.9)	60 (56.1)	47 (43.9)		
	Dental Hygienists	93	18 (19.3)	12 (66.7)	6 (33.3)	75 (80.7)	14 (18.6)	61 (81.4)		
Working hours per day	≤4 h	46	12 (26.1)	5 (41.6)	7 (58.4)	34 (73.9)	8 (23.6)	26 (76.4)	*p* = 0.040	*p* = 0.027
	5–8 h	145	23 (15.8)	13 (56.5)	10 (43.5)	122 (84.2)	54 (44.2)	68 (55.8)		
	≥8 h	93	8 (8.6)	6 (75.0)	2 (25.0)	85 (91.4)	35 (41.1)	50 (58.9)		
Working hours per week	12–20	32	8 (25.0)	2 (25.0)	6 (75.0)	24 (75.0)	6 (25.0)	18 (75.0)	*p* = 0.079	*p* = 0.045
	20–30	82	13 (15.8)	9 (69.2)	4 (30.8)	69 (84.2)	30 (43.4)	39 (56.6)		
	30–40	108	15 (13.8)	8 (53.3)	7 (46.7)	93 (86.2)	29 (31.2)	64 (68.8)		
	>40	62	7 (11.3)	5 (71.4)	2 (18.6)	55 (88.7)	32 (58.2)	23 (41.8)		
Years of work	Postgraduate students	44	10 (22.7)	7 (70.0)	3 (30.0)	34 (77.3)	6 (17.6)	28 (82.8)	*p* = 0.017	*p* = 0.284
	1 year	19	5 (26.3)	2 (40.0)	3 (60.0)	14 (73.6)	5 (35.7)	9 (64.3)		
	2–5 years	60	12 (20.0)	6 (50.0)	6 (50.0)	48 (80.0)	17 (35.4)	31 (64.6)		
	6–10 years	29	4 (13.7)	1 (25.0)	3 (75.0)	25 (86.3)	8 (32.0)	17 (68.0)		
	11–20 years	39	2 (5.1)	2 (100)	0	37 (94.9)	16 (43.2)	21 (57.8)		
	21–30 years	44	5 (11.3)	3 (60.0)	2 (40.0)	39 (88.7)	21 (53.8)	18 (46.2)		
	31–40 years	49	5 (10.2)	3 (60.0)	2 (40.0)	44 (89.8)	24 (54.4)	20 (45.6)		
Total		284	43 (15.1)	24 (55.8)	19 (44.2)	241 (84.9)	97 (40.2)	144 (59.8)	*p* = 0.048	

**Table 4:** WMSD in relation physical activity, their frequency and duration, mobilization exercises and knowledge of ergonomic guidelines (statistical significance for *p* < 0.05). All these parameters were not statistically significant (*p* > 0.05).

			Absence of WMSD			Presence of WMSD			χ^2^ Test	
			Total	Males	Females	Total	Males	Females	Males/Females	Total/Total
		*n*	*n* (%)	*n* (%)	*n* (%)	*n* (%)	*n* (%)	*n* (%)		
Physical activities	No	87	12 (13.8)	7 (58.3)	5 (41.7)	75 (86.2)	33 (44.0)	42 (55.0)	*p* = 0.350	*p* = 0.786
	Yes	197	31 (15.7)	17 (54.8)	14 (55.2)	166 (84.3)	64 (38.5)	102 (61.5)		
Physical activity duration(hours per week)	Never	83	11 (13.2)	7 (63.3)	4 (46.7)	72 (86.8)	31 (43.1)	41 (56.9)	*p* = 0.058	*p* = 0.897
	1 h	37	8 (21.6)	3 (37.5)	5 (62.5)	29 (78.4)	10 (34.4)	19 (65.6)		
	2–3 h	113	16 (14.5)	9 (65.2)	7 (34.8)	97 (85.5)	34 (35.1)	63 (64.9)		
	5 h	41	7 (17.7)	4 (57.1)	3 (42.9)	34 (82.3)	19 (55.8)	15 (44.2)		
	>10 h	10	1 (10)	1 (100)	0	9 (90)	3 (33.3)	6 (66.7)		
Physical activity frequency (time per week)	Never	82	11 (12.1)	7 (63.3)	4 (36.7)	71 (87.9)	31 (43.6)	40 (56.4)	*p* = 0.174	*p* = 0.763
	1/week	103	15 (14.5)	8 (53.3)	7 (46.7)	88 (85.5)	26 (29.5)	62 (70.5)		
	2–3/week	76	13 (17.1)	7 (53.8)	6 (46.2)	63 (82.9)	31 (49.2)	32 (50.8)		
	Every day	23	4 (17.4)	2 (50.0)	2 (50.0)	19 (82.6)	9 (47.3)	10 (52.7)		
Mobilization activities	Yes	202	31 (15.3)	20 (64.5)	11 (35.5)	171 (84.6)	64 (37.4)	107 (62.6)	*p* = 0.150	*p* = 0.879
	No	82	12 (14.7)	4 (33.3)	8 (66.7)	70 (85.3)	33 (47.1)	37 (52.9)		
Ergonomic knowledge	Yes	55	7 (12.7)	7 (100)	0	48 (87.2)	17 (35.4)	31 (64.6)	*p* = 0.243	*p* = 0.660
	No	127	22 (17.3)	9 (40.9)	13 (59.1)	105 (82.7)	38 (36.2)	67 (63.8)		
	Superficial	102	14 (13.7)	8 (57.1)	6 (42.9)	88 (86.3)	42 (47.7)	46 (52.3)		
Total		284	43 (15.1)	24 (55.8)	19 (44.2)	241 (84.9)	97 (40.2)	144 (59.8)	*p* = 0.048	

**Table 5:** Logistic regression of factors in association to WMSD. (statistical significance for *p* < 0.05). Gender and working hours per day parameters were significantly correlated to WMSD occurrence.

	Standard Error	Coefficient	*p*-Value	95.0% CI	
				Upper Boundary	Lower Boundary
Intercept	0.491	0.637	0.197	−0.332	1.606
Gender	0.070	0.152	0.031	0.014	0.289
Age	0.025	0.035	0.163	−0.014	0.084
Self-reported body fatness	0.050	0.078	0.126	−0.022	0.177
Height	0.031	0.018	0.557	−0.043	0.080
Occupation	0.007	−0.006	0.398	−0.019	0.007
Working hours per day	0.056	0.115	0.041	0.005	0.226
Working hours per week	0.040	−0.025	0.544	−0.104	0.055
Years of work	0.014	0.016	0.251	−0.012	0.044
Physical activity	0.217	0.153	0.483	−0.276	0.581
Physical activity duration	0.038	0.052	0.181	−0.024	0.127
Physical activity frequency	0.043	−0.012	0.781	−0.097	0.073
Ergonomic knowledge	0.036	0.025	0.491	−0.046	0.095
Mobilization activities	0.054	−0.005	0.922	−0.112	0.102

## Data Availability

The access to the data can be provided by the corresponding author upon signed declaration to respect confidentiality and data privacy.
